# Ultrasonographic Achilles Tendon Measurements and Static and Dynamic Balance in Prediabetes

**DOI:** 10.3390/medicina60081349

**Published:** 2024-08-19

**Authors:** Fulya Bakılan, Sultan Şan Kuşcu, Burcu Ortanca, Fezan Şahin Mutlu, Pınar Yıldız, Onur Armağan

**Affiliations:** 1 Department of Physical Medicine and Rehabilitation, Eskişehir Osmangazi University, 26040 Eskişehir, Turkey; sultann1905@gmail.com (S.Ş.K.); burcu-ayik@hotmail.com (B.O.); dronurarmagan@hotmail.com (O.A.); 2 Department of Biostatistics, Eskişehir Osmangazi University, 26040 Eskişehir, Turkey; fsahin@ogu.edu.tr; 3 Department of Internal Medicine, Eskişehir Osmangazi University, 26040 Eskişehir, Turkey; pinaresogu@gmail.com

**Keywords:** Achilles tendon, balance, dynamic balance, prediabetes, static balance

## Abstract

*Background and Objectives:* There is a lack of studies examining balance problems and Achilles tendon thickness in prediabetes despite their common occurrence in diabetes mellitus. The aim of this study was to evaluate Achilles tendon size and static and dynamic balance, as well as the role of the Achilles tendon in balance, in prediabetic patients. *Materials and Methods:* A total of 96 participants were divided into three groups: (1) the control group, consisting of participants without diabetes mellitus; (2) the prediabetes group; and (3) the diabetes mellitus group. Ultrasonographic measurements of Achilles tendon sizes (thickness, width and area) were performed. Dynamic balance was assessed using the Berg Balance Scale, and static balance (the Fall and Stability Indices) was assessed using a Tetrax device. The Self-Leeds Assessment of Neuropathic Symptoms and Signs was utilized to identify neuropathic pain. *Results:* In the prediabetes group, the median dynamic balance scores [54.0 (51.0–56.0)] were lower than those of the control group [55.0 (54.0–56.0)] but higher than those of the patients with diabetes mellitus [52.50 (49.0–54.25)]; however, this difference did not reach statistical significance. The ultrasonographic measurements of the Achilles tendon size were similar among the three groups. On the other hand, in the prediabetes group, a positive correlation was observed between the bilateral Achilles tendon anterior–posterior thickness and Fall Index score (*p* = 0.045), while a negative correlation was found between the left Achilles tendon anterior–posterior thickness and the Berg Balance Score (*p* = 0.045). *Conclusions:* In prediabetes, neither Achilles tendon size nor static or dynamic balance appears to be significantly affected. However, in prediabetic patients, increased Achilles tendon thickness appears to be associated with increased risk of falls and decreased balance.

## 1. Introduction

Multiple mechanisms contribute to controlling balance, including reactive, anticipatory, sensory and dynamic factors; the limits of the balance system; and physiological factors, such as the vestibular, visual and proprioceptive systems; muscle strength; reaction time; and the ankle and foot complex [1]. All these play crucial roles in maintaining balance [2]. Type 2 diabetes mellitus (DM) frequently induces changes that impact the somatosensory, vestibular and visual systems and lead to a high incidence of falls because of inadequate balance [3,4,5].

Previous studies have shown that patients with DM have had several changes in the ankle and foot complex [1,6], and the biomechanical characteristics of the ankle and foot complex have been reported to correlate with postural control in individuals with DM and be associated with vestibular inputs [1]. The Achilles tendon has been shown to be thicker in DM [1,7,8], while increases in the thickness of the Achilles tendon have been associated with the capacity of patients with DM to rely primarily on vestibular inputs for maintaining balance in the event of disrupted somatosensory input [1].

Prediabetes affects a wide section of the population and typically occurs for a significant period before the onset of DM. At the point of diagnosis, many patients already exhibit microvascular and macrovascular complications [9,10]. Individuals with prediabetes experience not only elevated peak glucose levels but also prolonged hyperglycemia due to their concurrent insulin resistance [11]. The latter triggers numerous metabolic processes, leading to endoneurial hypoxia and altering nerve perfusion, especially in glucose-dependent tissues such as the peripheral nerves and vestibular system. Furthermore, persistent hyperglycemia can result in muscle weakness, joint stiffness and premature degenerative alterations in the brain. On the other hand, it has been suggested that the thickening of the collagenous component, attributed to non-enzymatic glycation due to hyperglycemia, may be a factor contributing to the thickness of the Achilles tendon, which has been reported to be associated with vestibular inputs [1]. If persistent hyperglycemia disrupts balance through all the mechanisms described above, it is conceivable that impairment of balance and thickness of the Achilles tendon may also occur during the prediabetic period. Early detection of complications is important to reduce negative effects on patients’ quality of life not only in the DM stage but also at the prediabetic stage. In the literature, other musculoskeletal problems, such as carpal tunnel syndrome, have been reported to be more common in prediabetes [12]. Curiosity has been expressed about the Achilles tendon, particularly whether it is affected during the prediabetic period and its role in balance. However, to our knowledge, there has, as of now, been no study that has investigated balance, changes to the Achilles tendon or the role of the Achilles tendon in balance in the prediabetic period.

An emerging hypothesis states that Achilles tendon thickening and balance impairment may occur during the prediabetic period, which is considered to be the early stages of DM, and that the former may disrupt balance.

The primary aim of this study was to evaluate Achilles tendon size and static and dynamic balance in the prediabetic stage by comparing these factors in an appropriate group with those in the DM and control groups, while the secondary aim was to explore the correlation between the Achilles tendon size and static and dynamic balance.

## 2. Materials and Methods

### 2.1. Ethical Considerations

This trial was conducted in accordance with the ethical principles outlined in the 1964 Declaration of Helsinki, ensuring the protection of participants’ rights and well-being. Approval was obtained from the local ethics committee, with decision number 45, dated 17.01.23. Prior to participation, written informed consent was obtained from all participants after they were fully informed about this study’s purpose, procedures, potential risks and benefits. Ethical considerations, including the confidentiality of participant data and the voluntary nature of participation, were strictly upheld throughout this study.

### 2.2. Participants and Measurements

This cross-sectional study was conducted with 96 participants who were admitted to a Physical Medicine and Rehabilitation outpatient clinic between February and November 2023. They were divided into three groups: (1) the control group (those who were neither prediabetic nor diabetic; (2) the prediabetes group (prediabetic patients); and (3) the DM group (patients with Type 2 DM).

The demographic data of the participants were collected, including age, sex, body mass index (BMI) and duration of the disease. The laboratory data included HbA1c (%) and fasting plasma glucose (FPG) levels during the most recent three months.

Diagnosis of Type 2 DM was confirmed through patients’ medical records. Patients with an FPG of ≥126 mg/dL or 75 g oral glucose tolerance test that resulted in second-hour plasma glucose of ≥200 mg/dL and HbA1c ≥ 6.5% were included in the DM group.

Diagnosis of prediabetes was also confirmed through patients’ medical records. Patients with an FPG of 100–125 mg/dL or 75 g oral glucose tolerance test that resulted in second-hour plasma glucose of 140–199 mg/dL and HbA1c values of 5.7–6.4% were included in the prediabetes group.

The patients in the control group had an FPG of <100 mg/dL or 75 g oral glucose tolerance test that resulted in second-hour plasma glucose of <140 mg/dL and HbA1c values of <5.7% [13].

The final inclusion criterion for participation in this study was being between 40 and 65 years old. This age range was selected to focus on the adult population, as balance values can differ significantly between younger and geriatric populations.

Patients meeting any of the following criteria were excluded from this study: extremity amputation; vitamin B12 deficiency (all participants had documented B12 levels in the patient file system); Type 1 DM, prior exposure to neurotoxic agents; peripheral neuropathy for reasons such as chronic kidney failure, liver failure or hypothyroidism; hereditary or inflammatory peripheral neuropathies; neuromuscular diseases; malignancies; anti-neuropathic drug usage; radiculopathy; nerve trauma or surgery; vasculitis and autoimmune disorders; peripheral vascular disease; pregnancy; vestibular and cerebellar problems; history of lower extremity surgery; presence of medication affecting balance; history of alcoholism; and presence of visual impairment.

The Self-Leeds Assessment of Neuropathic Symptoms and Signs (S-LANSS) is a questionnaire consisting of seven items with Turkish validity and reliability. It is utilized to identify neuropathic pain. The S-LANSS was applied to all participants. The original S-LANSS, with a cut-off of ≥12, was considered indicative of the presence of neuropathic pain [14,15].

**Figure 1:**
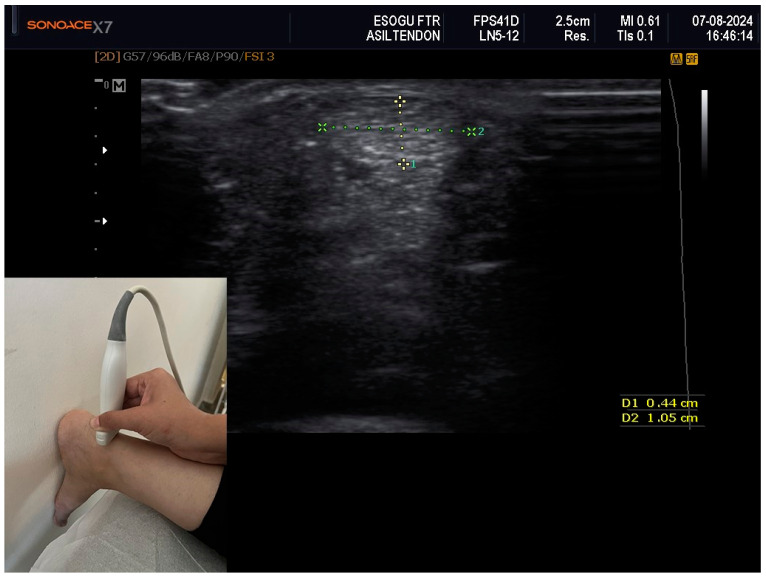
Ultrasound Imaging of the Achilles Tendon. The small image demonstrates the positioning of the ultrasound probe, placed perpendicular to the long axis of the Achilles tendon to obtain an axial plane at the level of the medial malleolus. The larger image depicts the measurements of the Achilles tendon, including thickness (anterior-posterior dimension, labeled as 1) and width (medial-lateral dimension, labeled as 2).

Ultrasonographic measurements of the Achilles tendon were taken using a Samsung Sonoace X7 ultrasound system (Samsung Medison Co., Ltd., Seoul, Republic of Korea) equipped with an 8–13 MHz linear transducer. After assuming a prone position on the examination table, each participant placed their feet against the wall and flexed their ankles to ensure optimal contact between the probe and the tendon. Measurements were taken separately on the right and left sides for each participant. Initially, the probe was positioned perpendicularly to the long axis of the tendon for an axial plane assessment, followed by measurements of the thickness (anterior–posterior), width (medial–lateral) and area at the level of the medial malleolus. The thickness and width measurements utilized the tendon’s major axes, while the area measurements were automatically calculated by the device through continuous tracing of the tendon circumference in the same section [16]. All sonographic measurements were performed by a sonographer with 5 years of experience in musculoskeletal ultrasound (Figure 1).

Dynamic balance was evaluated with the Berg Balance Test, which consists of 14 different questions evaluating the maintenance of static positions during changes in the center of body mass. Each participant was observed by a physician while performing the relevant activities, and the participants were given scores from 0 to 4. A score of 4 represented the activity being performed without any support, while 0 indicated the need for full support or the inability to perform the activity at all. The highest total score obtainable was 56, which represented excellent balance [17,18]. The test was performed only once per participant. To ensure accurate and reliable results, the following considerations were made: Participants were given sufficient rest before the test to avoid fatigue, which could have impacted balance performance. The test was conducted in a comfortable and distraction-free environment to facilitate optimal performance. Additionally, the purpose and instructions thereof were clearly explained to the participants to ensure they fully understood the procedure.

A Tetrax device (Tetrax-Sunlight Medical Ltd., Ramat Gan, Israel) was used to assess static balance. After the device was calibrated, each participant was positioned on the platform and subjected to tests in eight different positions. For each position, a test measurement was made for a duration of 32 s, totaling approximately 5 min. The normal eyes-open position was taken as a reference. The effects of vision on balance were observed in the eyes-closed position. When a participant was in the eyes-open position on a pillow, foam rubber pads limited the somatosensory system. In the eyes-closed position on a pillow, only the vestibular system functioned and was tested. With the head turned to the right and left in the eyes-closed position, both the vestibular and somatosensory systems were examined. In the eyes-closed position with the head tilted 30 degrees backward, the effects of central and peripheral vestibular disorders were observed. Balance was dependent on the back of the heels and the lower vertebrae in this position. Conversely, in the eyes-closed position with the head tilted 30 degrees forward, there was a load on the upper vertebrae and neck. Following the measurements, the same device was used to calculate the Fall and Stability Indices [19].

### 2.3. Statistical Analysis

A power analysis was conducted to determine the minimum required sample size for this study using the G*Power software, version 3.1.9.4 (Franz Faul, Universität Kiel, Düsseldorf, Germany). Based on the statistical findings from the reference publication, when the effect size (d) for the percentage change in the left Achilles tendon thickness parameter was taken as 5.0 and the standard deviation (SD) was taken as 0.1, the number of samples determined for power = 1.00 and α = 0.05 had minimums of *n*1= 22, *n*2 = 23 and *n*3 = 30 for each subgroup, respectively [20]. Considering the exclusion criteria, 30 participants were planned to be enrolled in each group.

In this study, the Shapiro–Wilk normality test was applied to the continuous variables. Descriptive statistics were presented as mean ± standard deviation (SD) and median (25th–75th percentiles), and categorical variables were presented as frequency and percentage. The independent samples *t*-test and one-way analysis of variance (ANOVA) were conducted for normally distributed variables. The Tukey and Tamhane Multiple Comparison tests were utilized to test differences among groups. The Mann–Whitney U and Kruskal–Wallis tests were applied for non-normally distributed variables, with Dunn’s Multiple Comparison Test used for intergroup differences. The Chi-square test was applied for categorical variables. The Spearman correlation coefficient was employed to examine correlation (a coefficient of <0.1: negligible correlation; 0.1–0.39: weak; 0.4–0.69: moderate; 0.7–0.89: strong; and ≥0.9: very strong) [21]. The significance level was set at *p* < 0.05. Statistical analyses were performed using the IBM SPSS Statistics 21.0 program (SPSS Inc., Chicago, IL, USA).

## 3. Results

Two patients were excluded due to chronic kidney failure, one patient was excluded due to a history of malignancy and three patients were excluded due to anti-neuropathic drug usage. Considering the inclusion and exclusion criteria, 90 patients, 72 females and 18 males, with a mean age of 53.06 ± 7.33, were accepted to our study.

The demographic characteristics of the three groups are shown in Table 1. Seven (23.3%) of the 30 patients with DM were using insulin, while twenty-eight (93.3%) of them were using oral anti-diabetic drugs. In the prediabetes group, only eight (26.7%) patients were using oral anti-diabetic drugs. None of the patients had a foot ulcer.

None of the control group participants, only one patient in the prediabetes group and only one patient in the DM group had an S-LANSS score greater than 12. This means that almost all the patients had an S-LANSS score lower than 12. A comparison of the static and dynamic balance parameters among the three groups showed that only the Berg Balance Score was significantly lower in the DM group than in the control group (*p* = 0.001) (Table 2). In the patients with prediabetes, the median dynamic balance scores [54.0 (51.0–56.0)] were lower than those of the control group [55.0 (54.0–56.0)] but higher than those of the patients with DM [52.50 (49.0–54.25)]; however, this difference did not reach statistical significance. Moreover, the ultrasonographic measurements of Achilles tendon size was similar among the three groups (Table 3).

In the DM group, a weak positive correlation was found between BMI and the bilateral Achilles tendon medio–lateral width (right side: *p* = 0.034, r = 0.388; left side: *p* = 0.042, r = 0.374). On the other hand, in the control group, a moderate negative correlation was found between BMI and Berg Balance Score values (*p* < 0.001, r = −0.658). In the prediabetes group, a moderate negative correlation was found among age (*p* = 0.002, r = −0.549), BMI (*p* = 0.003, r = −0.527) and the Berg Balance Score. In the same group, a weak positive correlation was observed among age, the bilateral Achilles tendon anterior–posterior thickness and the Fall Index score (right: *p* = 0.045, r = 0.368; left: *p* = 0.045, r = 0.369). Additionally, a weak negative correlation was found between the Berg Balance Score and the left Achilles tendon anterior–posterior thickness (*p* = 0.045, r = −0.369). In the DM group, a moderate negative correlation was observed between age and the Berg Balance Score (*p* = 0.025, r = −0.409), while a moderate positive correlation was observed between age and the Fall Index score (*p* = 0.007, r = 0.478) (Table 4).

## 4. Discussion

To the best of our knowledge, there is currently no documentation on whether the significant impairment of balance and Achilles tendon thickness observed in the diabetic stage are also present in the prediabetic stage.

The present study showed that in patients with prediabetes, the median dynamic balance scores were lower than those of the control group but higher than those of patients with DM; however, this difference did not reach statistical significance. This finding was consistent with the characteristic features of prediabetes, which typically include blood glucose levels that are above normal but below the thresholds for diabetes. Considering that the dynamic balance scores of the DM and prediabetes groups were found to be similar in our study, it cannot be concluded that dynamic balance is unimpaired in the prediabetes period. Further research with more sensitive assessment parameters is needed in this regard.

The static balance measurements, including the Fall and Stability Indices, with eyes both open and closed, were found to be similar across the three groups. Static balance did not appear to be affected in the patients with prediabetes or diabetes, who primarily presented without neuropathic pain. Nearly all the participants included in this study had an S-LANSS score below 12, indicating a minimal presence of neuropathic pain. This finding aligns with the existing literature. Previous studies have shown that neuropathy, which plays a role in the deterioration of balance, occurs not only in the diabetic stage but also in the prediabetic stages [22]. Palma et al. [23] conducted a comparison of static balance in Type 2 DM patients, both with and without neuropathy, and reported that static balance was worse in the former. In addition, Lim et al. [24] reported worse static balance scores in patients with diabetic polyneuropathy than in patients with diabetes but no polyneuropathy. In diabetic polyneuropathy, the presence of factors such as decreased proprioception, weakened muscles and reduced somatosensory function explains the deterioration of balance. If the S-LANSS scores of most of the patients in our DM and prediabetes groups had not been below 12, or if neuropathic patients had been predominant in our study, the static balance values of the diabetes and prediabetes groups could have been worse.

In the present study, neither the DM group nor the prediabetes group exhibited thickening of the Achilles tendon, which is the part of the ankle and foot complex that correlates to postural control and vestibular inputs [1]. Most of the Achilles tendon size parameters were higher in the prediabetes group than in the DM and control groups; however, this result did not reach statistical significance. If the number of participants in this study had been higher, we believe that a statistically significant difference could have been found. This result may have contributed to higher BMIs in prediabetes patients, as the increase in mechanical load on the foot, often observed in individuals with DM due to their higher body masses [25], is thought to play a role in the thickness of the Achilles tendon. In addition, BMI was positively correlated with Achilles tendon width in the DM group. In contrast to the findings of the present study, Achilles tendon thickness has been consistently associated with Type 2 DM in many studies [1,7,8]. Most have clarified this association by attributing it to neuropathy [8,26]. Evranos et al. [8] reported that Achilles tendon thickness values were significantly higher in diabetic patients with foot ulcers than in diabetic patients without foot ulcers and the control group. They also reported similar results to our findings between diabetic patients without foot ulcers and the control group. In our study, none of the patients had a foot ulcer, and the number of patients showing neuropathy symptoms was minimal.

In the literature, Achilles tendon size has been reported to be correlated with postural control in DM [1]. To our knowledge, this issue has not yet been investigated in prediabetes. In our study results, for the prediabetes patients, a positive correlation was observed between the bilateral Achilles tendon anterior–posterior thickness and the Fall Index score (which shows impaired static balance). Furthermore, in prediabetes patients, a negative correlation was found between the left Achilles tendon anterior–posterior thickness and the Berg Balance Score (which shows impaired dynamic balance). These results showed that not only age and BMI but also Achilles tendon thickness affect balance in the prediabetic stage. On the other hand, no correlation was found between static and dynamic balance and the Achilles tendon parameters in the control and DM groups. In DM, polypharmacy, muscle weakness of ankle dorsiflexion, and micro- and macrovascular complications, such as neuropathy and retinopathy [27], are all factors that affect balance and increase the risk of falls [28]. Without these other risk factors, the thickening of the Achilles tendon may not be significant or substantial enough to disrupt balance in overt diabetes by itself. However, in prediabetes, Achilles tendon thickness was found to affect both dynamic and static balance. Preventing the transition of patients from prediabetes to diabetes and ensuring that they adhere to the correct diets and exercise regimens are invaluable in preventing balance from being impaired. Monitoring Achilles tendon thickness using ultrasonography during the prediabetic period may provide guidance in monitoring balance.

## 5. Conclusions

The main limitation of this study is the absence of neuropathic diabetic and prediabetic groups due to the low S-LANSS scores of the patients participating in this study and the small sample size. The lack of assessment of dynamic balance using the Biodex Balance System or similar objective measurement tools is another. The biggest strength of the present study is that it is, to the best of our knowledge, the first study that has evaluated balance, Achilles tendon size and their relationship in the prediabetic stage. Balance was assessed both statically and dynamically, and moreover, static balance was measured using an objective tool. While the Berg Balance Scale offers advantages such as cost-effectiveness, ease of administration and minimal training requirements, its limitations, including ceiling effects and inadequate prediction of falls during active movement, may affect its ability to objectively measure balance in prediabetic patients. Therefore, more precise methods are needed to accurately assess balance in this population. Future research should focus on evaluating balance in prediabetic patients using advanced methodologies, such as electromyographic studies, to explore the relationship among balance, Achilles tendon size and neuropathy. Additionally, investigating alternative balance assessment tools and interventions could provide further insights into improving fall risk prediction and management in these patients.

In conclusion, in prediabetes, neither Achilles tendon size nor static or dynamic balance appears to be significantly affected. In prediabetic patients, increased Achilles tendon thickness appears to be associated with an increased risk of falls and decreased balance.

## Figures and Tables

**Table 1:** Demographic and laboratory data of all groups.

	Control Group *n* = 30 Median (25–75%)	Prediabetes Group *n* = 30 Median (25–75%)	DM Group *n* = 30 Median (25–75%)	*p*-Value
**Demographic data**				
Age (years)	52.0 (44.75–58.25)	55.0 (45.75–59.0)	56.0 (48.75–60.0)	0.495
Sex (Female/Male) *n* %	24 (80%)/6 (20%)	25 (83.3%)/5 (16.7%)	23 (76.7%)/7 (23.3%)	0.812
BMI ^1^ (kg/m^2^)	27.16 ± 4.11	31.67 ± 6.01 *	29.66 ± 4.18	**0.002**
Disease duration (months)	0 (0–0)	27.0 (3.0–48.0) ^a^	84.0 (36.0–171.0)	**<0.001**
**Laboratory data**				
HbA1c (%)	5.55 (5.37–5.60)	5.95 (5.77–6.10) °	7.0 (6.57–8.72)	**<0.001**
Fasting glucose levels (mg/dL)	89.5 (83.0–95.0)	96.0 (91.5–101.75) ^±^	133.0 (118.0–145.0)	**<0.001**

^1^ Mean ± SD (DM: Diabetes Mellitus). * The Prediabetes Group was significantly different from the Control Group (*p* = 0.004). ^a^ The Prediabetes Group was significantly different from the DM Group (*p* < 0.001). ° The Prediabetes Group was significantly different from the DM Group (*p* < 0.001) and the Control Group (*p* < 0.001). The DM Group was significantly different from the Control Group (*p* < 0.001). ^±^ The Prediabetes Group was significantly different from the DM Group (*p* < 0.001) and the Control Group (*p* = 0.019). The DM Group was significantly different from the Control Group (*p* < 0.001).

**Table 2:** Comparison of S-LANSS, the static and dynamic balance parameters among prediabetes, diabetes and control groups.

	Control Group *n* = 30 Median (25–75%)	Prediabetes Group *n* = 30 Median (25–75%)	DM Group *n* = 30 Median (25–75%)	*p*-Value
**S-LANSS ^1^**	0 (0–0)	0 (0–6) *	5.5 (1–8)	**<0.001**
**Static Balance**				
Stability Index (eyes open)	10.15 (8.77–12.37)	11.70 (9.15–14.42)	12.01 (10.45–15.77)	0.075
Stability Index (eyes closed)	17.10 (11.82–21.59)	17.85 (14.10–23.40)	19.25 (14.42–23.90)	0.251
Fall Index	21 (7.5–32.5)	24.0 (14.0–37.5)	21.0 (9.50–42.50)	0.624
**Dynamic Balance**				
Berg Balance Score	55.0 (54.0–56.0)	54.0 (51.0–56.0)	52.50 (49.0–54.25) °	**0.001**

^1^ S-LANSS: The Self-Leeds Assessment of Neuropathic Symptoms and Signs (DM: Diabetes Mellitus). * The Prediabetes Group was significantly different from the Control Group (*p* = 0.006) and the DM Group (*p* = 0.008). The DM Group was significantly different from the Control Group (*p* < 0.001). ° The DM Group was significantly different from the Control Group (*p* = 0.001).

**Table 3:** Comparison of the ultrasonographic measurement of the Achilles tendon among prediabetes, diabetes, and control groups.

	Control Group *n* = 30 Median (25–75%)	Prediabetes Group *n* = 30 Median (25–75%)	DM Group *n* = 30 Median (25–75%)	*p*-Value
**Ultrasonographic Measurements of Achilles Tendon (mm)**				
Thickness ^1^ (A-P) (R)	0.48 ± 0.08	0.50 ± 0.08	0.48 ± 0.07	0.547
Thickness (A-P) (L)	0.45 (0.40–0.50)	0.45 (0.41–0.53)	0.47 (0.43–0.50)	0.400
Width (M-L) (R)	1.24 (1.17–1.35)	1.31 (1.21–1.48)	1.30 (1.19–1.38)	0.327
Width ^1^ (M-L) (L)	1.28 ± 0.12	1.32 ± 0.17	1.28 ± 0.16	0.586
Area (R)	0.51 (0.44–0.60)	0.56 (0.46–0.70)	0.51 (0.47–0.58)	0.179
Area ^1^ (L)	0.51 ± 0.11	0.55 ± 0.13	0.53 ± 0.09	0.433

^1^ Mean ± SD, (DM: Diabetes Mellitus, R: Right, L: Left, A-P: Anterior–posterior, M-L: Medio–lateral, mm: millimeters).

**Table 4:** Correlation analysis among age, body mass index, ultrasonographic measurements of the Achilles tendon and static/dynamic balance scores.

	Control Group *n* = 30		Prediabetes Group *n* = 30		DM Group *n* = 30	
	Dynamic Balance (Berg Balance Score)	Static Balance (Fall Index)	Dynamic Balance (Berg Balance Score)	Static Balance (Fall Index)	Dynamic Balance (Berg Balance Score	Static Balance (Fall Index)
**Age (years)**	***p* = 0.122**	***p* = 0.372**	***p* = 0.002**	***p* = 0.038**	***p* = 0.025**	***p* = 0.007**
	**r = −0.288**	r = 0.169	**r = −0.549**	**r = 0.381**	**r = −0.409**	**r = 0.478**
**Body Mass Index (kg/m^2^)**	*p* < 0.001	*p* = 0.930	***p* = 0.003**	***p* = 0.181**	***p* = 0.840**	***p* = 0.456**
	r = −0.658	r = 0.017	**r = −0.527**	r = 0.251	r = −0.039	r = 0.141
**Ultrasonographic Measurements of Achilles Tendon (mm)**						
Thickness (A-P) (R)	***p* = 0.515**	*p* = 0.404	***p* = 0.330**	*p* = 0.045	*p* = 0.889	*p* = 0.774
	**r = −0.124**	r = 0.158	r = −0.184	**r = 0.368**	r = −0.027	r = 0.055
Thickness (A-P) (L)	*p* = 0.423	*p* = 0.676	*p* = 0.045	***p* = 0.045**	*p* = 0.902	*p* = 0.422
	r = −0.152	r = 0.080	**r = −0.369**	**r = 0.369**	r = −0.023	r = 0.152
Width (M-L) (R)	*p* = 0.388	*p* = 0.987	***p* = 0.394**	***p* = 0.594**	*p* = 0.925	*p* = 0.478
	r = −0.164	r = 0.003	r = 0.161	r = −0.101	r = −0.018	r = 0.135
Width (M-L) (L)	*p* = 0.297	*p* = 0.972	*p* = 0.593	*p* = 0.813	*p* = 0.595	*p* = 0.489
	r = −0.197	r = −0.007	r = 0.102	r = −0.045	r = −0.101	r = 0.131
Area (R)	*p* = 0.518	*p* = 0.707	*p* = 0.965	*p* = 0.527	*p* = 0.889	*p* = 0.889
	r = −0.123	r = 0.072	r = 0.008	r = 0.120	r = 0.027	r = 0.027
Area (L)	*p* = 0.503	*p* = 0.970	*p* = 0.874	*p* = 0.585	*p* = 0.736	*p* = 0.889
	r = −0.127	r = −0.007	r = −0.030	r = 0.104	r = −0.064	r = 0.027

DM: Diabetes Mellitus, R: Right, L: Left, A-P: Anterior–posterior, M-L: Medio–lateral, mm: millimeters.

## Data Availability

The data that support the findings of this study are available from the corresponding author upon reasonable request.

## References

[1] Cheing G.L., Chau R.M., Kwan R.L., Choi C.H., Zheng Y.P. (2013). Do the biomechanical properties of the ankle–foot complex influence postural control for people with Type 2 diabetes?. Clin. Biomech..

[2] Choi S., Jun H.-P. (2024). Effects of rehabilitative exercise and neuromuscular electrical stimulation on muscle morphology and dynamic balance in individuals with chronic ankle instability. Medicina.

[3] Tander B., Atmaca A., Ulus Y., Tura Ç., Akyol Y., Kuru Ö. (2016). Balance performance and fear of falling in older patients with diabetics: A comparative study with non-diabetic elderly. Turk. J. Phys. Med. Rehabil..

[4] Dixon C.J., Knight T., Binns E., Ihaka B., O’Brien D. (2017). Clinical measures of balance in people with type two diabetes: A systematic literature review. Gait Posture.

[5] Choi J.H., Kim H.R., Song K.H. (2022). Musculoskeletal complications in patients with diabetes mellitus. Korean J. Intern. Med..

[6] Tuna H., Birtane M., Güldiken S., Soysal N.A., Taşpinar Ö., Süt N., Taştekin N. (2014). The Effect of Disease Duration on Foot Plantar Pressure Values in Patients with Type 2 Diabetes Mellitus. Turk. J. Phys. Med. Rehabil..

[7] Papanas N., Courcoutsakis N., Papatheodorou K., Daskalogiannakis G., Maltezos E., Prassopoulos P. (2009). Achilles tendon volume in type 2 diabetic patients with or without peripheral neuropathy: MRI study. Exp. Clin. Endocrinol. Diabetes.

[8] Evranos B., Idilman I., Ipek A., Polat S.B., Cakir B., Ersoy R. (2015). Real-time sonoelastography and ultrasound evaluation of the Achilles tendon in patients with diabetes with or without foot ulcers: A cross sectional study. J. Diabetes Complicat..

[9] Baranowska-Jurkun A., Matuszewski W., Bandurska-Stankiewicz E. (2020). Chronic microvascular complications in prediabetic states—An overview. J. Clin. Med..

[10] Parakash S.S. (2023). Hyperinsulinemia, obesity, and diabetes mellitus. Int. J. Diabetes Dev. Ctries..

[11] Sharafi M., Eftekhari M.H., Mohsenpour M.A., Afrashteh S., Baeradeh N., Fararouei M., Pezeshki B. (2023). Progression of prediabetes to diabetes and its associated factors: The Fasa Adult Cohort Study (FACS). Int. J. Diabetes Dev. Ctries..

[12] Erol K., Topaloğlu U.S., Göl M.F. (2022). Frequency of carpal tunnel syndrome and hand dysfunction in prediabetes: A cross-sectional, controlled study. Turk. J. Phys. Med. Rehabil..

[13] (2024). American Diabetes Association Professional Practice Committee. 2. Diagnosis and Classification of Diabetes: Standards of Care in Diabetes—2024. Diabetes Care.

[14] Koç R., Erdemoglu A.K. (2010). Validity and reliability of the Turkish Self-administered Leeds Assessment of Neuropathic Symptoms and Signs (S-LANSS) questionnaire. Pain Med..

[15] Bennett M.I., Smith B.H., Torrance N., Potter J. (2005). The S-LANSS score for identifying pain of predominantly neuropathic origin: Validation for use in clinical and postal research. J. Pain.

[16] Canbolat M., Özbağ D., Özdemir Z., Demirtaş G., Kafkas A.Ş. (2015). Effects of physical characteristics, exercise and smoking on morphometry of human Achilles tendon: An ultrasound study. Anatomy.

[17] Berg K., Wood-Dauphinee S., Williams J.I. (1995). The Balance Scale: Reliability assessment with elderly residents and patients with an acute stroke. Scand. J. Rehabil. Med..

[18] Sahin F., Yilmaz F., Ozmaden A., Kotevoglu N., Sahin T., Kuran B. (2008). Reliability and validity of the Turkish version of the Berg Balance Scale. J. Geriatr. Phys. Ther..

[19] Bozbaş G.T., Gürer G. (2018). Does the lower extremity alignment affect the risk of falling?. Turk. J. Phys. Med. Rehabil..

[20] İyidir Ö.T., Rahatlı F.K., Bozkuş Y., Ramazanova L., Turnaoğlu H., Nar A., Tütüncü N.B. (2021). Acoustic radiation force impulse elastography and ultrasonographic findings of Achilles tendon in patients with and without diabetic peripheral neuropathy: A cross-sectional study. Exp. Clin. Endocrinol. Diabetes.

[21] Schober P., Boer C., Schwarte L.A. (2018). Correlation coefficients: Appropriate use and interpretation. Anesth. Analg..

[22] Koçer A., Domac F.M., Boylu E., Us Ö., Tanridağ T. (2007). A comparison of sural nerve conduction studies in patients with impaired oral glucose tolerance test. Acta Neurol. Scand..

[23] Palma F.H., Antigual D.U., Martínez S., Monrroy M.A., Gajardo R.E. (2013). Static balance in patients presenting diabetes mellitus type 2 with and without diabetic polyneuropathy. Arq. Bras. Endocrinol. Metab..

[24] Lim K.B., Kim D.J., Noh J.H., Yoo J., Moon J.W. (2014). Comparison of balance ability between patients with type 2 diabetes and with and without peripheral neuropathy. Pm&r.

[25] Duffin A.C., Lam A., Kidd R., Chan A.K.F., Donaghue K.C. (2002). Ultrasonography of plantar soft tissue thickness in young people with diabetes. Diabet. Med..

[26] Giacomozzi C., D’ambrogi E., Uccioli L., Macellari V. (2005). Does the thickening of Achilles tendon and plantar fascia contribute to the alteration of diabetic foot loading?. Clin. Biomech..

[27] Kafa N., Citaker S., Tuna Z., Guney H., Kaya D., Guzel N.A., Basar S., Yetkin I. (2015). Is plantar foot sensation associated with standing balance in type 2 diabetes mellitus patients. Int. J. Diabetes Dev. Ctries..

[28] Reeves N.D., Orlando G., Brown S.J. (2021). Sensory-motor mechanisms increasing falls risk in diabetic peripheral neuropathy. Medicina.

